# Q-Matrix Designs of Longitudinal Diagnostic Classification Models With Hierarchical Attributes for Formative Assessment

**DOI:** 10.3389/fpsyg.2020.01694

**Published:** 2020-07-30

**Authors:** Wei Tian, Jiahui Zhang, Qian Peng, Xiaoguang Yang

**Affiliations:** Collaborative Innovation Center of Assessment for Basic Education Quality, Beijing Normal University, Beijing, China

**Keywords:** Q-matrix, longitudinal DCMs, hierarchical attributes, TDCM, HDCM

## Abstract

Longitudinal diagnostic classification models (DCMs) with hierarchical attributes can characterize learning trajectories in terms of the transition between attribute profiles for formative assessment. A longitudinal DCM for hierarchical attributes was proposed by imposing model constraints on the transition DCM. To facilitate the applications of longitudinal DCMs, this paper explored the critical topic of the Q-matrix design with a simulation study. The results suggest that including the transpose of the R-matrix in the Q-matrix improved the classification accuracy. Moreover, 10-item tests measuring three linear attributes across three time points provided satisfactory classification accuracy for low-stakes assessment; lower classification rates were observed with independent or divergent attributes. Q-matrix design recommendations were provided for the short-test situation. Implications and future directions were discussed.

## Introduction

Diagnostic cognitive models (DCMs; or cognitive diagnostic models, CDMs) have received increasing attention because the latent variable modeling approach to diagnostic assessment can shed light on the learning process (Rupp et al., 2010). A variety of latent variable models have been proposed in recent decades including specific models (e.g., the Deterministic Input, Noisy “and” Gate, DINA; Junker and Sijtsma, 2001) and generalized frameworks (e.g., the log-linear cognitive diagnostic model, LCDM; Henson et al., 2009). Two recent directions aim to address hierarchical attributes (Gierl et al., 2010; Templin and Bradshaw, 2014) and the mastery of attributes in longitudinal data (Li et al., 2016; Kaya and Leite, 2017; Wang et al., 2017; Madison and Bradshaw, 2018a,b), respectively.

The transition DCM (TDCM), proposed by Madison and Bradshaw (2018a,b), is a longitudinal model combining the LCDM and the latent transition analysis (LTA). The TDCM have been used on tests measuring independent attributes (Madison and Bradshaw, 2018a,b). However, empirical studies have suggested the presence of interdependencies among attributes in many educational cases (e.g., Gierl et al., 2010; Templin and Bradshaw, 2014). The incorporation of attribute hierarchy into the Q-matrix and the model parameterization has become important research topics in recent years. One of the approaches to modeling the attribute relationships is to impose a hierarchical structure in which mastering an attribute could be a prerequisite to mastering another attribute (Tatsuoka, 1983; Leighton et al., 2004; Templin and Bradshaw, 2014). Taking this approach, Templin and Bradshaw (2014) extended LCDM to its hierarchical form—hierarchical diagnostic classification model (HDCM). Similarly, the longitudinal model TDCM can be constrained to incorporate hierarchical attributes. Following this line of thinking, we proposed the hierarchical transition DCM (H-TDCM) and explored the effects of Q-matrix designs on its classifications in this study.

The Q-matrix design, as a core element of the DCM-based test design, has not been adequately addressed in the context of longitudinal DCMs, since existing research focuses on model development and applications of longitudinal DCMs (e.g., Kaya and Leite, 2017; Madison and Bradshaw, 2018a,b). The Q-matrix links the items and the latent constructs to be measured (i.e., attributes) (Tatsuoka, 1983). Rows of the Q-matrix correspond to items, columns correspond to attributes, and its binary elements indicate whether an item measures an attribute (to put it differently, whether mastery of an attribute is required to succeed on an item). The row vectors of the Q-matrix are also called q-vectors. The Q-matrix plays important roles, both theoretically and statistically. From a theoretical perspective, cognitive theories could have a real impact on testing practice through the Q-matrix. This is especially true when the attributes are related to each other according to the cognitive theory. From a statistical perspective, the Q-matrix plays a significant role in model identification (Xu and Zhang, 2016; Xu, 2017; Köhn and Chiu, 2018; Gu and Xu, 2019a, forthcoming) and classification accuracy (DeCarlo, 2011; Madison and Bradshaw, 2015; Liu et al., 2017; Tu et al., 2019).

The identifiability conditions need to be satisfied for consistent estimation of the model parameters. Gu and Xu (2019a) identified the sufficient and necessary condition for identification of DINA and DINO. It requires that each attribute is measured by at least three items with a Q-matrix in the form $Q={\left(I_{K}^{T},{\left(Q^{\prime }\right)}^{T}\right)}^{T}$ (T denotes transpose), in which any two different columns of the submatrix *Q*′ are distinct (Gu and Xu, 2019a). The indentifiability issue is more complicated for saturated models (e.g., GDINA) and details on strict or generic identification can be found in Gu and Xu (forthcoming). The identification condition for hierarchical DCMs has also been discussed (Gu and Xu, forthcoming).

However, the Q-matrices that lead to identification may provide varying classification accuracy rates (DeCarlo, 2011; Madison and Bradshaw, 2015). To provide guidance for test construction practices based on DCMs, researchers explored the effects of different Q-matrix designs on the classification accuracy. For example, on the effects of Q-matrix designs with independent attributes, DeCarlo (2011) and Madison and Bradshaw (2015) have found that including more items measuring each attributes in isolation could help increase classification accuracy for DINA and LCDM.

**Figure 1:**
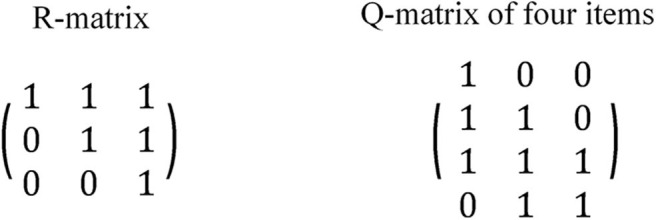
Example of R-matrix and Q-matrix for three linear attributes.

When attribute hierarchies are involved, there has not been a consensus on the Q-matrix design regarding whether all q-vectors are eligible (Templin and Bradshaw, 2014; Tu et al., 2019). When a test involves K independent attributes, there are 2^*K*^ − 1 distinct q-vectors. Consider a linear hierarchy with three attributes: α_1_ → α_2_ → α_3_. Attribute α_2_ has direct relationships with the other two attributes while Attribute α_1_ and α_3_ have an indirect relationship. The reachability matrix or R-matrix can be used to capture both direct and indirect relationships (Tatsuoka, 1983; Gierl et al., 2000; Leighton et al., 2004). The R-matrix for three attributes under a linear hierarchy is presented in Figure 1. Some researchers argued that an item cannot measure a higher-level attribute without measuring its prerequisite(s) (Leighton et al., 2004; Köhn and Chiu, 2018; Tu et al., 2019), referred to as the restricted Q-matrix approach. According to the restricted Q-matrix approach, only three q-vectors are allowed in the Q-matrix in the case of three linear attributes, which correspond to the three column vectors of the R-matrix. In contrast, some studies use all 2^*K*^ − 1 = 7 q-vectors in the Q-matrix as in an independent-attribute situation (Liu and Huggins-Manley, 2016; Liu et al., 2017), referred to as the unstructured Q-matrix approach.

Tu et al. (2019) took the restricted Q-matrix approach in a simulation study and emphasized the importance of containing the transpose of the R-matrix in the Q-matrix. Figure 1 provided an example Q-matrix containing the transpose of the R-matrix, *R*^*T*^. Liu et al. (2017), taking the unstructured Q-matrix approach, proposed different approaches to generate Q-matrices with linear, divergent, convergent, or unstructured attributes under the hierarchical diagnostic classification model (HDCM; Templin and Bradshaw, 2014). The adjacent approach (allowing each item to measure at most two attributes with direct relationships) was found to lead to higher classification accuracy in a shorter test (Liu et al., 2017).

To sum up, the purposes of the current study are 2-fold: First, the H-TDCM was defined to incorporate hierarchical attributes in the longitudinal DCM. Second, different Q-matrix designs were explored for TDCM and H-TDCM with a Monte Carlo simulation study. Both longitudinal models are based on LCDM, which is a general framework without limitations of the model fit assumptions. The rest of the paper is organized as follows. The next section briefly introduces LCDM, HDCM, and TDCM before defining the H-TDCM. Then, previous studies on the Q-matrix design are reviewed, followed by a simulation study on Q-matrix designs for TDCM and H-TDCM. The paper is concluded with a discussion of the limitations and educational implications.

## Models

### LCDM, HDCM, and TDCM

The LCDM (Henson et al., 2009) is a general diagnostic model that parameterizes the effects of the attributes measured by the item on the probability of a correct response given examinee attribute profile. The LCDM subsumes many specific DCMs, including the DINA model (Junker and Sijtsma, 2001) and the DINO model (Templin and Henson, 2006).

Examinee attribute profiles are denoted by vectors **α**_**c**_ = (α_*c*1_, …α_*ck*_, …, α_*cK*_), where *c* = 1, …, *C* and α_*ck*_ takes the value of 0 or 1, indicating the non-mastery or mastery, respectively, of the *k*th attribute. The LCDM classifies examinees into one of the *C* = 2^*K*^ attribute profiles assuming independent attributes. The number of attribute profiles decreases accordingly with hierarchical attributes.

For each item measured on a test, the LCDM item response function models the attributes mastery effects on the item response in terms of an intercept, the main effect for each attribute measured by the item, and the interaction term(s) that correspond to each possible combination of multiple attributes measured by the item. The general form of the LCDM item response function can be expressed as

(1) $$\begin{array}{l} P(X_{ic}=1|\alpha_{c})=\frac{\exp(\lambda_{i,0}+\;\lambda_{i}^{T}h(\alpha_{c},q_{i}))}{1+\exp(\lambda_{i,0}+\;\lambda_{i}^{T}h(\alpha_{c},q_{i}))} \end{array}$$

where λ_*i*,0_ is the intercept parameter of item *i*, **λ**_*i*_ contains all other item parameters including the main effects and interaction terms for item *i*, ***q***_*i*_ denotes the q-vector of item *i*, the superscript *T* denotes transpose, and the function **h** results in a linear combination of **α**_*c*_ and **q**_*i*_.

(2) $$\begin{array}{l} \lambda_{i,0}+\lambda_{i}^{T}h\left(\alpha_{c},q_{i}\right)=\lambda_{i,0}+\lambda_{i,1,\left(k\right)}\alpha_{ck}q_{ik}+\lambda_{i,2,(l(k))}\alpha_{ck}\alpha_{cl}q_{ik}q_{il}\\ \;+\lambda_{i,3,(m(l,k))}\alpha_{ck}\alpha_{cl}\alpha_{cm}q_{ik}q_{il}q_{im}+\ldots \end{array}$$

Templin and Bradshaw (2014) proposed the hierarchical diagnostic classification models (HDCM) to address hierarchical attributes. Specifically, two changes are made to LCDM. First, the attribute profile space is limited and **α**_*c*_ in Equations (1) and (2) is replaced by $\alpha_{c}^{*}$ for notation. When a linear hierarchy is assumed, the number of mastery profiles is reduced from the original *C* = 2^*K*^ to *C* = *K* + 1. The second change is that model constraints are imposed on LCDM. Specifically, some model parameters of the measurement model are fixed as zero.

Madison and Bradshaw (2018a,b) combined LCDM with latent transition analysis (LTA) to produce TDCM. LTA is a longitudinal latent class model that classifies examinees into latent classes and captures the latent class transitions over time (Collins and Lanza, 2010). As a conventional latent class analysis, it consists of the structural model and the measurement model. It is also a special case of the latent or hidden Markov model (HMM; Baum and Petrie, 1966). LTA parameterizes the probabilities of each latent class transitioning from one latent class to another between each time point in addition to latent class proportions and item parameters (i.e., the parameters estimated in conventional latent class analysis. LCDM serves as the measurement model of LTA. The LTA-DINA (Li et al., 2016) and LTA-DINO (Kaya et al., 2016) can be seen as special cases of the TDCM.

### H-TDCM

The proposed H-TDCM combined the features of HDCM and TDCM to deal with hierarchical attributes in longitudinal data. The attribute hierarchy is imposed on TDCM by constraining corresponding item parameters in the measurement model as in HDCM and the structural parameters that are specific to TDCM. Specifically, model parameters for the main effects of nested attributes and some interaction terms are constrained as zero in light of the prerequisite relationships among them. Also, similar constraints are set on the transition parameters and prevalence parameters.

Given the expression of LTA (Collins and Lanza, 2010, p. 198), the probability of an examinee's response vector on *I* items over *T* time points is given by

(3) $$\begin{array}{l} P\left(Y=y\right)=\overset{Structural}{\overset{︷}{\sum_{\alpha_{c_{1}}^{*}=1}^{C}\ldots \sum_{\alpha_{c_{T}}^{*}=1}^{C}\delta_{\alpha_{c_{1}}^{*}\;}\tau_{\alpha_{c_{2}|c_{1}}^{*}}\ldots \tau_{\alpha_{c_{T}|c_{T-1}}^{*}}}}\\ \overset{Measurement}{\overset{︷}{\prod_{t=1}^{T}\prod_{i=1}^{I}\prod_{r_{i,t}=1}^{R_{i}}{\left[\rho_{i,r_{i,t}|\alpha_{c_{t}}^{*},q_{i}}\right]}^{I\left(y_{i,t}=r_{i,t}\right)}}}\\ =\overset{Structural}{\overset{︷}{\sum_{\alpha_{c_{1}}^{*}=1}^{C}\ldots \sum_{\alpha_{c_{T}}^{*}=1}^{C}\delta_{\alpha_{c_{1}}^{*}\;}\tau_{\alpha_{c_{2}|c_{1}}^{*}}\ldots \tau_{\alpha_{c_{T}|c_{T-1}}^{*}}}}\\ \overset{Measurement}{\overset{︷}{\prod_{t=1}^{T}\prod_{i=1}^{I}\prod_{r_{i,t}=1}^{R_{i}}{\left[\frac{\exp(\lambda_{i,0}+\lambda_{i}^{T}h(\alpha_{c_{t}}^{*},q_{i}))}{1+exp(\lambda_{i,0}+\lambda_{i}^{T}h(\alpha_{c_{t}}^{*},q_{i}))}\;\right]}^{I\left(y_{i,t}=r_{i,t}\;\right)},}} \end{array}$$

where *i* = 1, 2, …, *I*; item *i* has *R*_*i*_ response categories; *y*_*i, t*_ is the examinee's response to item *i* at time point t and *I*(*y*_*i, t*_ = *r*_*i, t*_) is an indicator function that is equal to 1 when the response is *r*_*i, t*_, and equal to 0 otherwise; each sum ranges over each of the *C* attribute profiles at each time point, the first product is over the T time points, and the second product is over the I items; if the test measures *K* attributes with a certain hierarchical structure, the attribute profile at Time Point t is $\alpha_{c_{t}}^{*}=\left(\alpha_{1_{t}},\ldots ,\alpha_{k_{t}},\ldots ,\alpha_{K_{t}}\right)$, for simplicity, *C*_*t*_ = *C*.

There are three types of parameters to be estimated (similar to the case of TDCM) in Equation (3). The first type includes HDCM item parameters λ_*i*, 0_ and **λ**_*i*_. The second type is the probability of membership in attribute profile *c* at time point 1, denoted as δ_**α**_*c*_1___; and the third is the probability of transitioning between different attribute profiles (from **α**_*c*_*t*−1__ to **α**_*c*_*t*__) between time point t−1 to time point t, denoted as τ_**α**_*c*_*t*___**|α**_*c*_*t*−1__, usually expressed as a multinomial regression model (e.g., Reboussin et al., 1998; Nylund, 2007):

(4) $$\begin{array}{l} \tau_{\alpha_{c_{t}}|\alpha_{c_{t-1}}}=\frac{\exp(a_{c_{t}}+b_{c_{t}|c_{t-1}}^{T}d_{c_{t-1}})}{\sum_{c_{t}=1}^{C}\exp\left(a_{c_{t}}+b_{c_{t}|c_{t-1}}^{T}d_{c_{t-1}}\right)}\\ \;=\frac{\exp(a_{c_{t}}+b_{c_{t}|c_{t-1}}^{T}d_{c_{t-1}})}{1+\sum_{c_{t}=1}^{C-1}\exp\left(a_{c_{t}}+b_{c_{t}|c_{t-1}}^{T}d_{c_{t-1}}\right)},t\geq 2; \end{array}$$

We take for example a test measuring three linear attributes (α_1_ → α_2_ → α_3_). The *C* = 4 attribute profiles are the rows in

(5) $$\begin{array}{l} \left[\begin{array}{ccc} \alpha_{11} & \alpha_{12} & \alpha_{13}\\ \alpha_{21} & \alpha_{22} & \alpha_{23}\\ \alpha_{31} & \alpha_{32} & \alpha_{33}\\ \alpha_{41} & \alpha_{42} & \alpha_{43} \end{array}\right]=\left[\begin{array}{ccc} 0 & 0 & 0\\ 1 & 0 & 0\\ 1 & 1 & 0\\ 1 & 1 & 1 \end{array}\right]. \end{array}$$

Four item parameters are to be estimated including the intercept effect λ_*i*,0_, the main effect λ_*i*,1,(1)_, the second-order interaction effect λ_*i*,2,(2(1))_, and the third-order interaction effect λ_*i*,3,(3(2, 1))_:

(6) $$\begin{array}{l} \lambda_{i,0}+\lambda_{i}^{T}h\left(\alpha_{c}^{*},q_{i}\right)=\lambda_{i,0}+\lambda_{i,1,\left(1\right)}\alpha_{c1}q_{i1}+\lambda_{i,2,(2(1))}\alpha_{c1}\alpha_{c2}q_{i1}q_{i2}\\ \;+\lambda_{i,3,(3(2,1))}\alpha_{c1}\alpha_{c2}\alpha_{c3}q_{i1}q_{i2}q_{i3} \end{array}$$

Note that Equation (3) is a general form of the H-TDCM. The combination of LTA and any other specific hierarchical CDM can be realized by imposing parameter constraints. The H-TDCM, in turn, can be seen as a special case of TDCM, and the two models can be compared with a likelihood-ratio difference test (Collins and Lanza, 2010). When the attribute hierarchy exists, H-TDCM is supposed to provide a more succinct model with a better fit than TDCM (Templin and Bradshaw, 2014).

## Simulation Study

### Design

**Figure 2:**
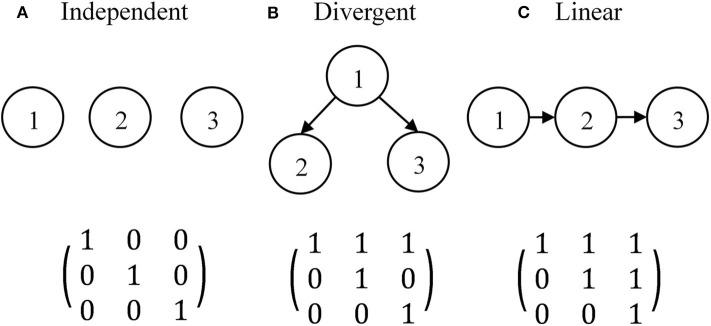
Three attribute hierarchies with three attributes and their R-matrix. **(A)** Independent. **(B)** Divergent. **(C)** Linear.

The simulation study aimed to explore the effects of different Q-matrices on the classifications of TDCM with or without an attribute hierarchy. There has been a need for short tests that measure a couple of fine-grained attributes in the classroom setting. The simulation conditions approximated a practical formative assessment over a learning period of 2–4 weeks. A limited number of attributes would be focused on within such a short period, and time for testing is also very limited so short sessions are preferred. This short test is supposed to be administered three times: at the beginning, in the middle, and approaching the end of the learning period. Therefore, the simulations only consider three-attribute tests administered over three time points. Three attribute hierarchies (independent, divergent, and linear) are considered. The three attribute hierarchies with three attributes and the associated R-matrices are presented in Figure 2.

As mentioned earlier, there are two general approaches to Q-matrix design with hierarchical attributes—the restricted and the unstructured Q-matrix approaches. The restricted Q-matrix approach only allows q-vectors in the transpose of the R-matrix, denoted as *R*^*T*^ (Leighton et al., 2004; Köhn and Chiu, 2018; Tu et al., 2019), and the general guideline is to contain several *R*^*T*^s in the Q-matrix to obtain acceptable classification accuracy (Tu et al., 2019). We took the unstructured Q-matrix approach, which means an item can measure all possible combinations of attributes as in an independent-attribute situation (Liu and Huggins-Manley, 2016; Liu et al., 2017), because there exists no empirical evidence against the possibility of items measuring a higher-level attribute without measuring its prerequisite(s). With three attributes in a test, there are seven q-vectors corresponding to seven item types. However, it remains an open question whether it is still beneficial to contain *R*^*T*^s in the Q-matrix even though the unstructured approach was adopted. For each attribute hierarchy, three Q-matrix designs were used. The first Q-matrix design does not contain *R*^*T*^, denoted as *Q*_1_. The second and third Q-matrix designs include one or two *R*^*T*^s, which are denoted as *Q*_2_ and *Q*_3_, respectively. Crossing two factors (i.e., attribute hierarchy and Q-matrix design) led to a total of 9 conditions. The simulation study focused on the Q-matrix design; thus, all Q-matrices were assumed to be correctly specified.

The item parameters are assumed to be time-invariant for the attribute profiles to retain the same meaning over time. Previous studies have shown that the examinee sample size barely has an impact on the classification rates of DCMs (de la Torre et al., 2010; Kaya and Leite, 2017). The effect of sample sizes was explored in Madison and Bradshaw (2018a) with TDCM. Therefore, the sample size was not manipulated but set to be 1,000 in each condition. The attribute profile of examinees followed a uniform distribution. Ten-item tests were generated under each condition.

To avoid the effects of item quality, we fixed the item parameters over all conditions: The intercept effect was −1, the main effect was 2, and the interaction effect was 1. As a result, *P*(*X* = 1|**α** = **0**) ranged from 0.1 to 0.3, and *P*(*X* = 1|**α** = **1**) was between 0.7 and 1.0. There are 8, 5, and 4 attribute profiles under independent, divergent, and linear hierarchies, respectively. With three independent attributes, there were 2^3^ attribute profiles: *c*_1_ (0, 0, 0), *c*_2_ (0, 0, 1), *c*_3_ (0, 1, 0), *c*_4_ (0, 1, 1), *c*_5_ (1, 0, 0), *c*_6_ (1, 0, 1), *c*_7_ (1, 1, 0), and *c*_8_ (1, 1, 1). The divergent hierarchy condition had *c*_1_ (0, 0, 0), *c*_5_ (1, 0, 0), *c*_6_ (1, 0, 1), *c*_7_ (1, 1, 0), and *c*_8_ (1, 1, 1). Three linear attributes led to four attribute profiles: *c*_1_ (0, 0, 0), *c*_5_ (1, 0, 0), *c*_7_ (1, 1, 0), and *c*_8_ (1, 1, 1).

Mplus 7.4 (Muthén and Muthén, 1998–2015) was used to generate and analyze the response data of three time points based on TDCM or H-TCDM via maximum likelihood estimation. We include the Mplus syntax for estimation as an Supplementary Material. Evaluation criteria include the marginal correct classification rates (MCCRs) for each attribute and the correct classification rates (CCRs) for each attribute profile. Each simulation condition was replicated 100 times.

## Results

The correct classification rates are presented in Table 1. The results suggested that including the transpose of the R-matrix in the Q-matrix (i.e., *Q*_2_) increased the profile CCRs and marginal CCRs at each time point for independent, divergent, and linear hierarchies. Including one more transpose of the R-matrix (i.e., *Q*_3_) further slightly increased the CCRs except for the linear hierarchy. Another interesting finding is that the profile CCRs tended to increase with time. The CCRs at Time 3 were the highest. This trend was found under each combination of attribute hierarchy and Q-matrix design. The increase with time was not found in the marginal CCRs for independent attributes. Within the divergent or linear hierarchy, the marginal CCRs of the highest-level attribute (i.e., α_2_ and α_3_ under the divergent hierarchy and α_3_ under the linear hierarchy) increased with time while the lowest-level attribute (i.e., α_1_) had decreasing CCRs with time.

**Table 1:** Classification rates of three Q-matrix designs.

	**Independent**			**Divergent**			**Linear**		
	**Q1**	**Q2**	**Q3**	**Q1**	**Q2**	**Q3**	**Q1**	**Q2**	**Q3**
**PROFILE CORRECT CLASSIFICATION RATES**									
Time 1	0.517	0.550	0.557	0.582	0.651	0.671	0.710	0.731	0.725
Time 2	0.522	0.553	0.556	0.595	0.667	0.681	0.725	0.749	0.736
Time 3	0.536	0.577	0.577	0.606	0.680	0.693	0.734	0.761	0.744
Mean	0.525	0.560	0.563	0.594	0.666	0.682	0.723	0.747	0.735
**MARGINAL CORRECT CLASSIFICATION RATES**									
Time 1 α_1_	0.723	0.784	0.821	0.938	0.937	0.917	0.931	0.929	0.901
Time 1 α_2_	0.833	0.838	0.809	0.714	0.795	0.840	0.864	0.887	0.904
Time 1 α_3_	0.831	0.807	0.810	0.855	0.858	0.860	0.864	0.872	0.885
Mean	0.796	0.810	0.813	0.836	0.863	0.872	0.886	0.896	0.897
Time 2 α_1_	0.704	0.774	0.809	0.929	0.925	0.903	0.915	0.913	0.881
Time 2 α_2_	0.827	0.835	0.803	0.716	0.804	0.845	0.848	0.875	0.890
Time 2 α_3_	0.828	0.796	0.798	0.857	0.859	0.864	0.927	0.932	0.937
Mean	0.787	0.802	0.804	0.834	0.863	0.871	0.897	0.907	0.903
Time 3 α_1_	0.713	0.784	0.816	0.927	0.924	0.901	0.912	0.912	0.877
Time 3 α_2_	0.835	0.840	0.806	0.724	0.811	0.852	0.849	0.876	0.890
Time 3 α_3_	0.834	0.807	0.808	0.864	0.864	0.868	0.944	0.947	0.950
Mean	0.794	0.810	0.810	0.838	0.866	0.874	0.902	0.912	0.906

*Three Q-matrix designs Q_1_, Q_2_, and Q_3_ included zero, one, or two R-matrix transposes*.

Comparing the three attribute hierarchies revealed that the CCRs generally increased as the relationship between attributes became stronger, and meanwhile, the number of attribute profiles became smaller. The profile CCRs were above 0.7, and the marginal CCRs were above 0.85 under the linear hierarchy with 10-item tests. The classifications for the independent attributes were the most difficult.

## Discussion

This paper proposed H-TDCM for hierarchical attributes in the longitudinal DCM by imposing model constraints on TDCM. The simulation study explored Q-matrix designs with different numbers of R-matrices. The CCRs generally increased with stronger dependencies between attributes, which is consistent with the findings of Templin and Bradshaw (2014) with LCDM. Ten-item tests for three linear attributes lead to profile CCRs above 0.7 and marginal CCRs above 0.85 at each time point, which might to acceptable for low-stakes classroom assessment. However, longer tests are needed for independent or divergent attributes to obtain acceptable classification rates. The profile CCRs increased with time, which means the attribute profile estimate from the final test would be the most accurate among several tests. The final attribute profile estimation may benefit from information from all the previous tests and provides a relatively accurate picture of the learning outcome, which is a desirable property for the longitudinal model.

Regarding the Q-matrix design, we took the unstructured Q-matrix approach (Liu and Huggins-Manley, 2016; Liu et al., 2017) by allowing all possible q-vectors, but explored Q-matrix designs containing different numbers of *R*^*T*^. Simulation results showed that including one R-matrix transpose in the Q-matrix increased the CCRs in the case of independent attributes. Note that although the identification issue of CDMs and the Q-matrix design are usually treated as two separate research areas, the identification requirement may not always be satisfied in the Q-matrix design studies, especially for more complicated models and shorter tests.

First, we looked at the results for independent attributes. A closer look at the Q-matrices revealed that the first Q-matrix design (*Q*_1_) did not measure α_1_ in isolation; the second Q-matrix design (*Q*_2_) contained only one identity matrix and measured α_1_ in isolation only once. This explained the much lower classification rates for α_1_ compared with other attributes. This finding with the TDCM agrees with the results of conventional DCMs (DeCarlo, 2011; Madison and Bradshaw, 2015). From the identification perspective, it has been proven that including two identity matrices in the Q-matrix is necessary for a saturated DCM such as LCDM with independent attributes (Gu and Xu, forthcoming). Under *Q*_1_ and *Q*_2_ for independent attributes, the model parameters suffered from the non-identifiability issue and the consequence was reflected in the lower profile CCRs with *Q*_1_ and *Q*_2_ than with *Q*_3_ in Table 1. It also explains why the marginal CCRs of α_1_ under *Q*_1_ and *Q*_2_ were substantially lower than those under *Q*_3_, while the marginal CCRs of the other two attributes did not differ much between Q-matrix designs.

Including *R*^*T*^ in the Q-matrix also increases the classification rates for the hierarchical cases in this study, which is consistent with the empirical findings from Tu et al. (2019). The results for hierarchical attributes can also be explained from the identification perspective as discussed in Gu and Xu (forthcoming). For a generalized multi-parameter DCM such as LCDM or HDCM, the concept of a separable Γ -matrix was introduced (Gu and Xu, forthcoming). The rows and columns of the Γ -matrix is indexed by the items and the attribute profiles, respectively. An entry of the Γ -matrix equals to 1 if an attribute profile has the highest correct response probability on an item and 0 otherwise. A Γ -matrix is said to be separable if any two column vectors of are distinct. The separability of the Γ -matrix is necessary for strict identification. We show that *R*^*T*^ as a submatrix in the Q-matrix ensures a separable Γ -matrix in Table 2. It can be further shown that the matrix of *R*^*T*^ is in the form of

$$\begin{array}{l} {\left(\begin{array}{llll} 1 & * & \cdots & *\\ {}* & 1 & \cdots & *\\ \vdots & \vdots & \ddots & \vdots \\ {}* & * & \cdots & 1 \end{array}\right)}_{K\times K} \end{array}$$

after some row permutation, in which ^*^ takes the value of 0 or 1 and K is the number of attributes. Two *R*^*T*^s were contained in *Q*_3_, which led to a separable Γ -matrix. As a result, *Q*_3_ always ensures the identification of the model, while the first design may lead to non-identification issues (Gu and Xu, forthcoming). In contrast, *Q*_2_ contained one *R*^*T*^ and at least one identity matrix instead of two *R*^*T*^s, which does not affect the model identification. Therefore, *Q*_2_ and *Q*_3_ showed similar classification rates. One major difference between the two designs is that *Q*_2_ contains more single-attribute items and fewer multiple-attribute items. Under the linear hierarchy, for example, *Q*_3_ has at least two items with q=(111), which has seven item parameters to be estimated. The parameter recovery of such items may be more difficult than single-attribute items, and the classification rate may suffer. As a result, the performance of *Q*_2_ turned out to be better than *Q*_3_ for the linear hierarchy.

**Table 2:** *R*^*T*^ as a submatrix in the Q-matrix ensures a separable Γ -matrix.

**q-vector**	**Attribute profile**			
	**000**	**100**	**110**	**111**
100	0	1	1	1
110	0	0	1	1
111	0	0	0	1

This study aimed to demonstrate the classification performance of the H-TDCM with a short test and provide practical guidelines for the applications of this longitudinal model for formative classroom assessment. For the current setting of short tests and only a few attributes, we recommend that the Q-matrix contains (1) two identity matrices for independent attributes, (2) two *R*^*T*^s for a divergent hierarchy, and (3) one *R*^*T*^ and one identity matrix for a linear hierarchy. Besides, each attribute should be probed by at least three items. However, it should be noted that the current simulation study assumes that it is possible to develop items of all types of q-vectors with equal easiness, which may not be true for certain subject areas. For example, it may be more difficult to develop items that measure each attribute in isolation.

The formative classroom assessment has received renewed attention recently with the development of curriculum reform. The fusion of curriculum, instruction, and the assessment requires timely and constructive feedback that is closely connected to a curriculum and are based on students' learning history (e.g., Bennett, 2015; Gotwals, 2018; Shepard et al., 2018). Such feedback can be obtained from a diagnostic model that portrays the progression of attribute profiles. To establish the learning progression in terms of attribute profiles, however, is not an easy task. A possible solution could be collecting longitudinal assessment data from multiple classrooms and applying H-TDCM. The model parameters and classification results from H-TDCM can be used to understand the learning process better and to give teachers and students prior information before the learning begins. The current study focused on short tests for classroom applications where the attribute hierarchy is prespecified. Future simulation research can extend to longer tests for the purpose of exploring the learning process by estimating the attribute hierarchy. Those who are interested may refer to the requirement on the Q-matrix design (Gu and Xu, 2019b).

## Data Availability Statement

The datasets generated for this study are available on request to the corresponding author.

## Author Contributions

All authors listed have made a substantial, direct and intellectual contribution to the work, and approved it for publication.

## Conflict of Interest

The authors declare that the research was conducted in the absence of any commercial or financial relationships that could be construed as a potential conflict of interest.
