# Supplementary material for: Q-Matrix Designs of Longitudinal Diagnostic Classification Models With Hierarchical Attributes for Formative Assessment
Source: Front Psychol. 2020 Jul 30;11:1694. doi: 10.3389/fpsyg.2020.01694 (PMC7438705; doi:10.3389/fpsyg.2020.01694)
Supplement: Supplementary file 1 [file Table_1.DOCX]

Example Mplus syntax

TITLE:

H-TDCM for DATA with 3 attributes and 3-order structural model,

10 items, average item parameters and maximum 3-order item model,Saturated structural model (Mplus default).

DATA: FILE IS data.dat;

VARIABLE:

NAMES = mitemf1-mitemf10 mitems1-mitems10 mitemt1-mitemt10;

USEVARIABLE = mitemf1-mitemf10 mitems1-mitems10 mitemt1-mitemt10;

CATEGORICAL = mitemf1-mitemf10 mitems1-mitems10 mitemt1-mitemt10;

CLASSES = c1(8) c2(8) c3(8);

ANALYSIS:

TYPE = MIXTURE;

MODEL:

%OVERALL%

!Set the class probabilities at the first time

[C1#1*0] (m1); !latent variable mean for attribute pattern [0,0,0];

[C1#2*0] (m2); !latent variable mean for attribute pattern [0,0,1];

[C1#3*0] (m3); !latent variable mean for attribute pattern [0,1,0];

[C1#4*0] (m4); !latent variable mean for attribute pattern [0,1,1];

[C1#5*0] (m5); !latent variable mean for attribute pattern [1,0,0];

[C1#6*0] (m6); !latent variable mean for attribute pattern [1,0,1];

[C1#7*0] (m7); !latent variable mean for attribute pattern [1,1,0];

!Set the transition probabilities from time 1 to time 2

!C2 ON C1,alpha(intercept)

[C2#1*0] (1);

[C2#2*0] (2);

[C2#3*0] (3);

[C2#4*0] (4);

[C2#5*0] (5);

[C2#6*0] (6);

[C2#7*0] (7);

C2#1 ON C1#1*2 (8);

C2#1 ON C1#2*2 (9);

C2#1 ON C1#3*2 (10);

C2#1 ON C1#4*2 (11);

C2#1 ON C1#5*2 (12);

C2#1 ON C1#6*2 (13);

C2#1 ON C1#7*2 (14);

C2#2 ON C1#1*2 (15);

C2#2 ON C1#2*2 (16);

C2#2 ON C1#3*2 (17);

C2#2 ON C1#4*2 (18);

C2#2 ON C1#5*2 (19);

C2#2 ON C1#6*2 (20);

C2#2 ON C1#7*2 (21);

C2#3 ON C1#1*2 (22);

C2#3 ON C1#2*2 (23);

C2#3 ON C1#3*2 (24);

C2#3 ON C1#4*2 (25);

C2#3 ON C1#5*2 (26);

C2#3 ON C1#6*2 (27);

C2#3 ON C1#7*2 (28);

C2#4 ON C1#1*2 (29);

C2#4 ON C1#2*2 (30);

C2#4 ON C1#3*2 (31);

C2#4 ON C1#4*2 (32);

C2#4 ON C1#5*2 (33);

C2#4 ON C1#6*2 (34);

C2#4 ON C1#7*2 (35);

C2#5 ON C1#1*2 (36);

C2#5 ON C1#2*2 (37);

C2#5 ON C1#3*2 (38);

C2#5 ON C1#4*2 (39);

C2#5 ON C1#5*2 (40);

C2#5 ON C1#6*2 (41);

C2#5 ON C1#7*2 (42);

C2#6 ON C1#1*2 (43);

C2#6 ON C1#2*2 (44);

C2#6 ON C1#3*2 (45);

C2#6 ON C1#4*2 (46);

C2#6 ON C1#5*2 (47);

C2#6 ON C1#6*2 (48);

C2#6 ON C1#7*2 (49);

C2#7 ON C1#1*2 (50);

C2#7 ON C1#2*2 (51);

C2#7 ON C1#3*2 (52);

C2#7 ON C1#4*2 (53);

C2#7 ON C1#5*2 (54);

C2#7 ON C1#6*2 (55);

C2#7 ON C1#7*2 (56);

!Set the transition probabilities from time 2 to time 3

!C3 ON C2,alpha(intercept)

[C3#1*0] (1);

[C3#2*0] (2);

[C3#3*0] (3);

[C3#4*0] (4);

[C3#5*0] (5);

[C3#6*0] (6);

[C3#7*0] (7);

C3#1 ON C2#1*2 (8);

C3#1 ON C2#2*2 (9);

C3#1 ON C2#3*2 (10);

C3#1 ON C2#4*2 (11);

C3#1 ON C2#5*2 (12);

C3#1 ON C2#6*2 (13);

C3#1 ON C2#7*2 (14);

C3#2 ON C2#1*2 (15);

C3#2 ON C2#2*2 (16);

C3#2 ON C2#3*2 (17);

C3#2 ON C2#4*2 (18);

C3#2 ON C2#5*2 (19);

C3#2 ON C2#6*2 (20);

C3#2 ON C2#7*2 (21);

C3#3 ON C2#1*2 (22);

C3#3 ON C2#2*2 (23);

C3#3 ON C2#3*2 (24);

C3#3 ON C2#4*2 (25);

C3#3 ON C2#5*2 (26);

C3#3 ON C2#6*2 (27);

C3#3 ON C2#7*2 (28);

C3#4 ON C2#1*2 (29);

C3#4 ON C2#2*2 (30);

C3#4 ON C2#3*2 (31);

C3#4 ON C2#4*2 (32);

C3#4 ON C2#5*2 (33);

C3#4 ON C2#6*2 (34);

C3#4 ON C2#7*2 (35);

C3#5 ON C2#1*2 (36);

C3#5 ON C2#2*2 (37);

C3#5 ON C2#3*2 (38);

C3#5 ON C2#4*2 (39);

C3#5 ON C2#5*2 (40);

C3#5 ON C2#6*2 (41);

C3#5 ON C2#7*2 (42);

C3#6 ON C2#1*2 (43);

C3#6 ON C2#2*2 (44);

C3#6 ON C2#3*2 (45);

C3#6 ON C2#4*2 (46);

C3#6 ON C2#5*2 (47);

C3#6 ON C2#6*2 (48);

C3#6 ON C2#7*2 (49);

C3#7 ON C2#1*2 (50);

C3#7 ON C2#2*2 (51);

C3#7 ON C2#3*2 (52);

C3#7 ON C2#4*2 (53);

C3#7 ON C2#5*2 (54);

C3#7 ON C2#6*2 (55);

C3#7 ON C2#7*2 (56);

MODEL c1:

%c1#1% ! Model for Class 1

[mitemf1$1*1] (T1_1); ! Item 1 Thresh 1

[mitemf2$1*1] (T2_1); ! Item 2 Thresh 1

[mitemf3$1*1] (T3_1); ! Item 3 Thresh 1

[mitemf4$1*1.5] (T4_1); ! Item 4 Thresh 1

[mitemf5$1*1.5] (T5_1); ! Item 5 Thresh 1

[mitemf6$1*1.5] (T6_1); ! Item 6 Thresh 1

[mitemf7$1*2] (T7_1); ! Item 7 Thresh 1

[mitemf8$1*1] (T8_1); ! Item 8 Thresh 1

[mitemf9$1*1] (T9_1); ! Item 9 Thresh 1

[mitemf10$1*1] (T10_1); ! Item 10 Thresh 1

%c1#2% ! Model for Class 2

[mitemf1$1*1] (T1_1); ! Item 1 Thresh 1

[mitemf2$1*1] (T2_1); ! Item 2 Thresh 1

[mitemf3$1*-1.5] (T3_2); ! Item 3 Thresh 2

[mitemf4$1*1.5] (T4_1); ! Item 4 Thresh 1

[mitemf5$1*0.5] (T5_2); ! Item 5 Thresh 2

[mitemf6$1*0.5] (T6_2); ! Item 6 Thresh 2

[mitemf7$1*1.5] (T7_2); ! Item 7 Thresh 2

[mitemf8$1*1] (T8_1); ! Item 8 Thresh 1

[mitemf9$1*1] (T9_1); ! Item 9 Thresh 1

[mitemf10$1*-1.5] (T10_2); ! Item 10 Thresh 2

%c1#3% ! Model for Class 3

[mitemf1$1*1] (T1_1); ! Item 1 Thresh 1

[mitemf2$1*-1.5] (T2_2); ! Item 2 Thresh 2

[mitemf3$1*1] (T3_1); ! Item 3 Thresh 1

[mitemf4$1*0.5] (T4_2); ! Item 4 Thresh 2

[mitemf5$1*1.5] (T5_1); ! Item 5 Thresh 1

[mitemf6$1*0.5] (T6_3); ! Item 6 Thresh 3

[mitemf7$1*1.5] (T7_3); ! Item 7 Thresh 3

[mitemf8$1*1] (T8_1); ! Item 8 Thresh 1

[mitemf9$1*-1.5] (T9_2); ! Item 9 Thresh 2

[mitemf10$1*1] (T10_1); ! Item 10 Thresh 1

%c1#4% ! Model for Class 4

[mitemf1$1*1] (T1_1); ! Item 1 Thresh 1

[mitemf2$1*-1.5] (T2_2); ! Item 2 Thresh 2

[mitemf3$1*-1.5] (T3_2); ! Item 3 Thresh 2

[mitemf4$1*0.5] (T4_2); ! Item 4 Thresh 2

[mitemf5$1*0.5] (T5_2); ! Item 5 Thresh 2

[mitemf6$1*-3] (T6_4); ! Item 6 Thresh 4

[mitemf7$1*-0.8] (T7_4); ! Item 7 Thresh 4

[mitemf8$1*1] (T8_1); ! Item 8 Thresh 1

[mitemf9$1*-1.5] (T9_2); ! Item 9 Thresh 2

[mitemf10$1*-1.5] (T10_2); ! Item 10 Thresh 2

%c1#5% ! Model for Class 5

[mitemf1$1*-1.5] (T1_2); ! Item 1 Thresh 2

[mitemf2$1*1] (T2_1); ! Item 2 Thresh 1

[mitemf3$1*1] (T3_1); ! Item 3 Thresh 1

[mitemf4$1*0.5] (T4_3); ! Item 4 Thresh 3

[mitemf5$1*0.5] (T5_3); ! Item 5 Thresh 3

[mitemf6$1*1.5] (T6_1); ! Item 6 Thresh 1

[mitemf7$1*1.5] (T7_5); ! Item 7 Thresh 5

[mitemf8$1*-1.5] (T8_2); ! Item 8 Thresh 2

[mitemf9$1*1] (T9_1); ! Item 9 Thresh 1

[mitemf10$1*1] (T10_1); ! Item 10 Thresh 1

%c1#6% ! Model for Class 6

[mitemf1$1*-1.5] (T1_2); ! Item 1 Thresh 2

[mitemf2$1*1] (T2_1); ! Item 2 Thresh 1

[mitemf3$1*-1.5] (T3_2); ! Item 3 Thresh 2

[mitemf4$1*0.5] (T4_3); ! Item 4 Thresh 3

[mitemf5$1*-3] (T5_4); ! Item 5 Thresh 4

[mitemf6$1*0.5] (T6_2); ! Item 6 Thresh 2

[mitemf7$1*-0.8] (T7_6); ! Item 7 Thresh 6

[mitemf8$1*-1.5] (T8_2); ! Item 8 Thresh 2

[mitemf9$1*1] (T9_1); ! Item 9 Thresh 1

[mitemf10$1*-1.5] (T10_2); ! Item 10 Thresh 2

%c1#7% ! Model for Class 7

[mitemf1$1*-1.5] (T1_2); ! Item 1 Thresh 2

[mitemf2$1*-1.5] (T2_2); ! Item 2 Thresh 2

[mitemf3$1*1] (T3_1); ! Item 3 Thresh 1

[mitemf4$1*-3] (T4_4); ! Item 4 Thresh 4

[mitemf5$1*0.5] (T5_3); ! Item 5 Thresh 3

[mitemf6$1*0.5] (T6_3); ! Item 6 Thresh 3

[mitemf7$1*-0.8] (T7_7); ! Item 7 Thresh 7

[mitemf8$1*-1.5] (T8_2); ! Item 8 Thresh 2

[mitemf9$1*-1.5] (T9_2); ! Item 9 Thresh 2

[mitemf10$1*1] (T10_1); ! Item 10 Thresh 1

%c1#8% ! Model for Class 8

[mitemf1$1*-1.5] (T1_2); ! Item 1 Thresh 2

[mitemf2$1*-1.5] (T2_2); ! Item 2 Thresh 2

[mitemf3$1*-1.5] (T3_2); ! Item 3 Thresh 2

[mitemf4$1*-3] (T4_4); ! Item 4 Thresh 4

[mitemf5$1*-3] (T5_4); ! Item 5 Thresh 4

[mitemf6$1*-3] (T6_4); ! Item 6 Thresh 4

[mitemf7$1*-4] (T7_8); ! Item 7 Thresh 8

[mitemf8$1*-1.5] (T8_2); ! Item 8 Thresh 2

[mitemf9$1*-1.5] (T9_2); ! Item 9 Thresh 2

[mitemf10$1*-1.5] (T10_2); ! Item 10 Thresh 2

MODEL c2:

%c2#1% ! Model for Class 1

[mitems1$1*1] (T1_1); ! Item 1 Thresh 1

[mitems2$1*1] (T2_1); ! Item 2 Thresh 1

[mitems3$1*1] (T3_1); ! Item 3 Thresh 1

[mitems4$1*1.5] (T4_1); ! Item 4 Thresh 1

[mitems5$1*1.5] (T5_1); ! Item 5 Thresh 1

[mitems6$1*1.5] (T6_1); ! Item 6 Thresh 1

[mitems7$1*2] (T7_1); ! Item 7 Thresh 1

[mitems8$1*1] (T8_1); ! Item 8 Thresh 1

[mitems9$1*1] (T9_1); ! Item 9 Thresh 1

[mitems10$1*1] (T10_1); ! Item 10 Thresh 1

%c2#2% ! Model for Class 2

[mitems1$1*1] (T1_1); ! Item 1 Thresh 1

[mitems2$1*1] (T2_1); ! Item 2 Thresh 1

[mitems3$1*-1.5] (T3_2); ! Item 3 Thresh 2

[mitems4$1*1.5] (T4_1); ! Item 4 Thresh 1

[mitems5$1*0.5] (T5_2); ! Item 5 Thresh 2

[mitems6$1*0.5] (T6_2); ! Item 6 Thresh 2

[mitems7$1*1.5] (T7_2); ! Item 7 Thresh 2

[mitems8$1*1] (T8_1); ! Item 8 Thresh 1

[mitems9$1*1] (T9_1); ! Item 9 Thresh 1

[mitems10$1*-1.5] (T10_2); ! Item 10 Thresh 2

%c2#3% ! Model for Class 3

[mitems1$1*1] (T1_1); ! Item 1 Thresh 1

[mitems2$1*-1.5] (T2_2); ! Item 2 Thresh 2

[mitems3$1*1] (T3_1); ! Item 3 Thresh 1

[mitems4$1*0.5] (T4_2); ! Item 4 Thresh 2

[mitems5$1*1.5] (T5_1); ! Item 5 Thresh 1

[mitems6$1*0.5] (T6_3); ! Item 6 Thresh 3

[mitems7$1*1.5] (T7_3); ! Item 7 Thresh 3

[mitems8$1*1] (T8_1); ! Item 8 Thresh 1

[mitems9$1*-1.5] (T9_2); ! Item 9 Thresh 2

[mitems10$1*1] (T10_1); ! Item 10 Thresh 1

%c2#4% ! Model for Class 4

[mitems1$1*1] (T1_1); ! Item 1 Thresh 1

[mitems2$1*-1.5] (T2_2); ! Item 2 Thresh 2

[mitems3$1*-1.5] (T3_2); ! Item 3 Thresh 2

[mitems4$1*0.5] (T4_2); ! Item 4 Thresh 2

[mitems5$1*0.5] (T5_2); ! Item 5 Thresh 2

[mitems6$1*-3] (T6_4); ! Item 6 Thresh 4

[mitems7$1*-0.8] (T7_4); ! Item 7 Thresh 4

[mitems8$1*1] (T8_1); ! Item 8 Thresh 1

[mitems9$1*-1.5] (T9_2); ! Item 9 Thresh 2

[mitems10$1*-1.5] (T10_2); ! Item 10 Thresh 2

%c2#5% ! Model for Class 5

[mitems1$1*-1.5] (T1_2); ! Item 1 Thresh 2

[mitems2$1*1] (T2_1); ! Item 2 Thresh 1

[mitems3$1*1] (T3_1); ! Item 3 Thresh 1

[mitems4$1*0.5] (T4_3); ! Item 4 Thresh 3

[mitems5$1*0.5] (T5_3); ! Item 5 Thresh 3

[mitems6$1*1.5] (T6_1); ! Item 6 Thresh 1

[mitems7$1*1.5] (T7_5); ! Item 7 Thresh 5

[mitems8$1*-1.5] (T8_2); ! Item 8 Thresh 2

[mitems9$1*1] (T9_1); ! Item 9 Thresh 1

[mitems10$1*1] (T10_1); ! Item 10 Thresh 1

%c2#6% ! Model for Class 6

[mitems1$1*-1.5] (T1_2); ! Item 1 Thresh 2

[mitems2$1*1] (T2_1); ! Item 2 Thresh 1

[mitems3$1*-1.5] (T3_2); ! Item 3 Thresh 2

[mitems4$1*0.5] (T4_3); ! Item 4 Thresh 3

[mitems5$1*-3] (T5_4); ! Item 5 Thresh 4

[mitems6$1*0.5] (T6_2); ! Item 6 Thresh 2

[mitems7$1*-0.8] (T7_6); ! Item 7 Thresh 6

[mitems8$1*-1.5] (T8_2); ! Item 8 Thresh 2

[mitems9$1*1] (T9_1); ! Item 9 Thresh 1

[mitems10$1*-1.5] (T10_2); ! Item 10 Thresh 2

%c2#7% ! Model for Class 7

[mitems1$1*-1.5] (T1_2); ! Item 1 Thresh 2

[mitems2$1*-1.5] (T2_2); ! Item 2 Thresh 2

[mitems3$1*1] (T3_1); ! Item 3 Thresh 1

[mitems4$1*-3] (T4_4); ! Item 4 Thresh 4

[mitems5$1*0.5] (T5_3); ! Item 5 Thresh 3

[mitems6$1*0.5] (T6_3); ! Item 6 Thresh 3

[mitems7$1*-0.8] (T7_7); ! Item 7 Thresh 7

[mitems8$1*-1.5] (T8_2); ! Item 8 Thresh 2

[mitems9$1*-1.5] (T9_2); ! Item 9 Thresh 2

[mitems10$1*1] (T10_1); ! Item 10 Thresh 1

%c2#8% ! Model for Class 8

[mitems1$1*-1.5] (T1_2); ! Item 1 Thresh 2

[mitems2$1*-1.5] (T2_2); ! Item 2 Thresh 2

[mitems3$1*-1.5] (T3_2); ! Item 3 Thresh 2

[mitems4$1*-3] (T4_4); ! Item 4 Thresh 4

[mitems5$1*-3] (T5_4); ! Item 5 Thresh 4

[mitems6$1*-3] (T6_4); ! Item 6 Thresh 4

[mitems7$1*-4] (T7_8); ! Item 7 Thresh 8

[mitems8$1*-1.5] (T8_2); ! Item 8 Thresh 2

[mitems9$1*-1.5] (T9_2); ! Item 9 Thresh 2

[mitems10$1*-1.5] (T10_2); ! Item 10 Thresh 2

MODEL c3:

%c3#1% ! Model for Class 1

[mitemt1$1*1] (T1_1); ! Item 1 Thresh 1

[mitemt2$1*1] (T2_1); ! Item 2 Thresh 1

[mitemt3$1*1] (T3_1); ! Item 3 Thresh 1

[mitemt4$1*1.5] (T4_1); ! Item 4 Thresh 1

[mitemt5$1*1.5] (T5_1); ! Item 5 Thresh 1

[mitemt6$1*1.5] (T6_1); ! Item 6 Thresh 1

[mitemt7$1*2] (T7_1); ! Item 7 Thresh 1

[mitemt8$1*1] (T8_1); ! Item 8 Thresh 1

[mitemt9$1*1] (T9_1); ! Item 9 Thresh 1

[mitemt10$1*1] (T10_1); ! Item 10 Thresh 1

%c3#2% ! Model for Class 2

[mitemt1$1*1] (T1_1); ! Item 1 Thresh 1

[mitemt2$1*1] (T2_1); ! Item 2 Thresh 1

[mitemt3$1*-1.5] (T3_2); ! Item 3 Thresh 2

[mitemt4$1*1.5] (T4_1); ! Item 4 Thresh 1

[mitemt5$1*0.5] (T5_2); ! Item 5 Thresh 2

[mitemt6$1*0.5] (T6_2); ! Item 6 Thresh 2

[mitemt7$1*1.5] (T7_2); ! Item 7 Thresh 2

[mitemt8$1*1] (T8_1); ! Item 8 Thresh 1

[mitemt9$1*1] (T9_1); ! Item 9 Thresh 1

[mitemt10$1*-1.5] (T10_2); ! Item 10 Thresh 2

%c3#3% ! Model for Class 3

[mitemt1$1*1] (T1_1); ! Item 1 Thresh 1

[mitemt2$1*-1.5] (T2_2); ! Item 2 Thresh 2

[mitemt3$1*1] (T3_1); ! Item 3 Thresh 1

[mitemt4$1*0.5] (T4_2); ! Item 4 Thresh 2

[mitemt5$1*1.5] (T5_1); ! Item 5 Thresh 1

[mitemt6$1*0.5] (T6_3); ! Item 6 Thresh 3

[mitemt7$1*1.5] (T7_3); ! Item 7 Thresh 3

[mitemt8$1*1] (T8_1); ! Item 8 Thresh 1

[mitemt9$1*-1.5] (T9_2); ! Item 9 Thresh 2

[mitemt10$1*1] (T10_1); ! Item 10 Thresh 1

%c3#4% ! Model for Class 4

[mitemt1$1*1] (T1_1); ! Item 1 Thresh 1

[mitemt2$1*-1.5] (T2_2); ! Item 2 Thresh 2

[mitemt3$1*-1.5] (T3_2); ! Item 3 Thresh 2

[mitemt4$1*0.5] (T4_2); ! Item 4 Thresh 2

[mitemt5$1*0.5] (T5_2); ! Item 5 Thresh 2

[mitemt6$1*-3] (T6_4); ! Item 6 Thresh 4

[mitemt7$1*-0.8] (T7_4); ! Item 7 Thresh 4

[mitemt8$1*1] (T8_1); ! Item 8 Thresh 1

[mitemt9$1*-1.5] (T9_2); ! Item 9 Thresh 2

[mitemt10$1*-1.5] (T10_2); ! Item 10 Thresh 2

%c3#5% ! Model for Class 5

[mitemt1$1*-1.5] (T1_2); ! Item 1 Thresh 2

[mitemt2$1*1] (T2_1); ! Item 2 Thresh 1

[mitemt3$1*1] (T3_1); ! Item 3 Thresh 1

[mitemt4$1*0.5] (T4_3); ! Item 4 Thresh 3

[mitemt5$1*0.5] (T5_3); ! Item 5 Thresh 3

[mitemt6$1*1.5] (T6_1); ! Item 6 Thresh 1

[mitemt7$1*1.5] (T7_5); ! Item 7 Thresh 5

[mitemt8$1*-1.5] (T8_2); ! Item 8 Thresh 2

[mitemt9$1*1] (T9_1); ! Item 9 Thresh 1

[mitemt10$1*1] (T10_1); ! Item 10 Thresh 1

%c3#6% ! Model for Class 6

[mitemt1$1*-1.5] (T1_2); ! Item 1 Thresh 2

[mitemt2$1*1] (T2_1); ! Item 2 Thresh 1

[mitemt3$1*-1.5] (T3_2); ! Item 3 Thresh 2

[mitemt4$1*0.5] (T4_3); ! Item 4 Thresh 3

[mitemt5$1*-3] (T5_4); ! Item 5 Thresh 4

[mitemt6$1*0.5] (T6_2); ! Item 6 Thresh 2

[mitemt7$1*-0.8] (T7_6); ! Item 7 Thresh 6

[mitemt8$1*-1.5] (T8_2); ! Item 8 Thresh 2

[mitemt9$1*1] (T9_1); ! Item 9 Thresh 1

[mitemt10$1*-1.5] (T10_2); ! Item 10 Thresh 2

%c3#7% ! Model for Class 7

[mitemt1$1*-1.5] (T1_2); ! Item 1 Thresh 2

[mitemt2$1*-1.5] (T2_2); ! Item 2 Thresh 2

[mitemt3$1*1] (T3_1); ! Item 3 Thresh 1

[mitemt4$1*-3] (T4_4); ! Item 4 Thresh 4

[mitemt5$1*0.5] (T5_3); ! Item 5 Thresh 3

[mitemt6$1*0.5] (T6_3); ! Item 6 Thresh 3

[mitemt7$1*-0.8] (T7_7); ! Item 7 Thresh 7

[mitemt8$1*-1.5] (T8_2); ! Item 8 Thresh 2

[mitemt9$1*-1.5] (T9_2); ! Item 9 Thresh 2

[mitemt10$1*1] (T10_1); ! Item 10 Thresh 1

%c3#8% ! Model for Class 8

[mitemt1$1*-1.5] (T1_2); ! Item 1 Thresh 2

[mitemt2$1*-1.5] (T2_2); ! Item 2 Thresh 2

[mitemt3$1*-1.5] (T3_2); ! Item 3 Thresh 2

[mitemt4$1*-3] (T4_4); ! Item 4 Thresh 4

[mitemt5$1*-3] (T5_4); ! Item 5 Thresh 4

[mitemt6$1*-3] (T6_4); ! Item 6 Thresh 4

[mitemt7$1*-4] (T7_8); ! Item 7 Thresh 8

[mitemt8$1*-1.5] (T8_2); ! Item 8 Thresh 2

[mitemt9$1*-1.5] (T9_2); ! Item 9 Thresh 2

[mitemt10$1*-1.5] (T10_2); ! Item 10 Thresh 2

MODEL CONSTRAINT: ! Used to define LCDM parameters

!STRUCTURAL MODEL:

NEW(g_0 g_11 g_12 g_13 g_212 g_213 g_223 g_3123);

m1=-(g_11+g_12+g_13+g_212+g_213+g_223+g_3123);

m2=g_13-(g_11+g_12+g_13+g_212+g_213+g_223+g_3123);

m3=g_12-(g_11+g_12+g_13+g_212+g_213+g_223+g_3123);

m4=g_12+g_13+g_223-(g_11+g_12+g_13+g_212+g_213+g_223+g_3123);

m5=g_11-(g_11+g_12+g_13+g_212+g_213+g_223+g_3123);

m6=g_11+g_13+g_213-(g_11+g_12+g_13+g_212+g_213+g_223+g_3123);

m7=g_11+g_12+g_212-(g_11+g_12+g_13+g_212+g_213+g_223+g_3123);

g_0=-(g_11+g_12+g_13+g_212+g_213+g_223+g_3123);

! Mplus uses P(X=0) rather than P(X=1) so multiply by -1

! Item 1: Define LCDM parameters present for item 1

NEW(L1_0 L1_11);

T1_1=-(L1_0); ! Item 1 Thresh 1

T1_2=-(L1_0+L1_11); ! Item 1 Thresh 2

! Main effect order constraints

L1_11>0;

! Item 2: Define LCDM parameters present for item 2

NEW(L2_0 L2_12);

T2_1=-(L2_0); ! Item 2 Thresh 1

T2_2=-(L2_0+L2_12); ! Item 2 Thresh 2

! Main effect order constraints

L2_12>0;

! Item 3: Define LCDM parameters present for item 3

NEW(L3_0 L3_13);

T3_1=-(L3_0); ! Item 3 Thresh 1

T3_2=-(L3_0+L3_13); ! Item 3 Thresh 2

! Main effect order constraints

L3_13>0;

! Item 4: Define LCDM parameters present for item 4

NEW(L4_0 L4_11 L4_12 L4_212);

T4_1=-(L4_0); ! Item 4 Thresh 1

T4_2=-(L4_0+L4_12); ! Item 4 Thresh 2

T4_3=-(L4_0+L4_11); ! Item 4 Thresh 3

T4_4=-(L4_0+L4_11+L4_12+L4_212); ! Item 4 Thresh 4

! Main effect order constraints

L4_11>0; L4_12>0;

! Two-way interaction order constraints

L4_212>-L4_11;

L4_212>-L4_12;

! Item 5: Define LCDM parameters present for item 5

NEW(L5_0 L5_11 L5_13 L5_213);

T5_1=-(L5_0); ! Item 5 Thresh 1

T5_2=-(L5_0+L5_13); ! Item 5 Thresh 2

T5_3=-(L5_0+L5_11); ! Item 5 Thresh 3

T5_4=-(L5_0+L5_11+L5_13+L5_213); ! Item 5 Thresh 4

! Main effect order constraints

L5_11>0; L5_13>0;

! Two-way interaction order constraints

L5_213>-L5_11;

L5_213>-L5_13;

! Item 6: Define LCDM parameters present for item 6

NEW(L6_0 L6_12 L6_13 L6_223);

T6_1=-(L6_0); ! Item 6 Thresh 1

T6_2=-(L6_0+L6_13); ! Item 6 Thresh 2

T6_3=-(L6_0+L6_12); ! Item 6 Thresh 3

T6_4=-(L6_0+L6_12+L6_13+L6_223); ! Item 6 Thresh 4

! Main effect order constraints

L6_12>0; L6_13>0;

! Two-way interaction order constraints

L6_223>-L6_12;

L6_223>-L6_13;

! Item 7: Define LCDM parameters present for item 7

NEW(L7_0 L7_11 L7_12 L7_13 L7_212 L7_213 L7_223 L7_3123);

T7_1=-(L7_0); ! Item 7 Thresh 1

T7_2=-(L7_0+L7_13); ! Item 7 Thresh 2

T7_3=-(L7_0+L7_12); ! Item 7 Thresh 3

T7_4=-(L7_0+L7_12+L7_13+L7_223); ! Item 7 Thresh 4

T7_5=-(L7_0+L7_11); ! Item 7 Thresh 5

T7_6=-(L7_0+L7_11+L7_13+L7_213); ! Item 7 Thresh 6

T7_7=-(L7_0+L7_11+L7_12+L7_212); ! Item 7 Thresh 7

T7_8=-(L7_0+L7_11+L7_12+L7_13+L7_212+L7_213+L7_223+L7_3123); ! Item 7 Thresh 8

! Main effect order constraints

L7_11>0; L7_12>0; L7_13>0;

! Two-way interaction order constraints

L7_212>-L7_11; L7_213>-L7_11; L7_223>-L7_12;

L7_212>-L7_12; L7_213>-L7_13; L7_223>-L7_13;

! Three-way interaction order constraints

L7_3123>-(L7_223+L7_213+L7_13);

L7_3123>-(L7_223+L7_212+L7_12);

L7_3123>-(L7_213+L7_212+L7_11);

! Item 8: Define LCDM parameters present for item 8

NEW(L8_0 L8_11);

T8_1=-(L8_0); ! Item 8 Thresh 1

T8_2=-(L8_0+L8_11); ! Item 8 Thresh 2

! Main effect order constraints

L8_11>0;

! Item 9: Define LCDM parameters present for item 9

NEW(L9_0 L9_12);

T9_1=-(L9_0); ! Item 9 Thresh 1

T9_2=-(L9_0+L9_12); ! Item 9 Thresh 2

! Main effect order constraints

L9_12>0;

! Item 10: Define LCDM parameters present for item 10

NEW(L10_0 L10_13);

T10_1=-(L10_0); ! Item 10 Thresh 1

T10_2=-(L10_0+L10_13); ! Item 10 Thresh 2

! Main effect order constraints

L10_13>0;

OUTPUT:

TECH1 TECH8 TECH10;! TECH10 Request additional model fit statistics

SAVEDATA:

FILE=ltalcdm1.dat;

SAVE = CPROBABILITIES;
